# The role of bladder instillation in the treatment of bladder pain syndrome: Is intravesical treatment an effective option for patients with bladder pain as well as LUTS?

**DOI:** 10.1007/s00192-020-04303-7

**Published:** 2020-05-01

**Authors:** Giuseppe Alessandro Digesu, Visha Tailor, Alka A. Bhide, Vik Khullar

**Affiliations:** St Mary’s Hospital, Imperial College NHS Trust, London, UK

**Keywords:** Bladder pain, Interstitial cystitis, Bladder instillations

## Abstract

The aetiology of bladder pain syndrome/interstitial cystitis is still unknown. Numerous mechanisms have been proposed and treatments targeting various aspects of these are used. This review looks at the existing evidence on bladder instillations and whether they could be used in the treatment of lower urinary tract symptoms as well.

## Introduction

We all know from our medical studies that any sensation that humans feel is the result of a stimulation of the peripheral sensory afferent nerves. The sensory stimuli travel towards the central nervous system and brain, which translate it into a sensation. Therefore, a dysfunctional somatic nerve system plays an important role in the pathogenesis of the lower urinary tract symptoms (LUTS) including pain reported by the patients. It has also been proposed that a local inflammatory process involving both afferent and efferent nerves in the suburothelial interstitial cellular network, which integrates the transmission of signals from the urothelium to the detrusor muscles in the bladder wall, is one of the main key factors in the pathogenesis of bladder pain syndrome (BPS)/interstitial cystitis (IC).

There is strong evidence that BPS/IC, as well as overactive bladder (OAB) symptoms, may be part of the same bladder disorder. It has been postulated that both OAB and BPS/IC share the same mechanism, which includes:

1. An alteration of the urothelial barrier due to a urothelial injury such as urinary tract infection, surgical trauma, chronic bladder overdistention
2. Suburothelial inflammation
3. Chronic inflammatory cell infiltration in the suburothelium with activation of mast cells
4. An increased inflammatory reaction in the sensory afferents, dorsal horn ganglia, and corresponding spinal cord (neurogenic inflammation)

Therefore, it has been speculated that LUTS such as pain and OAB can be treated by: repairing a damaged thin denudated urothelium, as well as treating the suburothelial inflammation and desensitising irritated sensory nerves. BPS/IC causes significant morbidity, with patients finding the condition debilitating, unpredictable and unrelenting and often results in them feeling isolated [1, 2].

If we all accept the idea that an inflammation of the bladder wall is the key factor in the development of LUTS, what causes the inflammation in the first place is still unknown. Many different aetiologies have been proposed including:

1. A post-infection autoimmune process
2. Mast cell activation induced by toxins, or stress
3. Urothelial dysfunction with increased permeability
4. Neurogenic inflammation

However, none of these aetiologies has been definitely proven [3].

## The role of the urothelium

It is well established that the urothelium plays an important role in communicating with the bladder nerves, smooth muscle, immune cells and inflammatory systems. The urothelium of the urinary bladder is covered by a glycosaminoglycans (GAGs) layer, which acts as a barrier between urine with its solutes and the underlying bladder wall. GAGs are extremely hydrophilic and trap water at the outer layer of the umbrella cell. A disruption of the GAG barrier may initiate a cascade of events in the bladder, leading to the migration of urinary solutes, in particular, potassium into the suburothelium, which depolarises nerves as well as muscles, causing tissue injury, which is responsible for LUTS and pain. Therefore, a leaky epithelium and subsequent neurogenic inflammation has been suggested as the primary cause of LUTS.

## Bladder inflammation and LUTS

Any insult to the urothelium or directly to the bladder wall may induce a cascade of inflammatory reactions and produce painful inflammation as well several other LUTS. It has been suggested that LUTS are the result of a long-standing inflammation of the bladder.

Chronic inflammation is implicated in the development of both OAB and BPS/IC. Elevation of serum C-reactive protein (CRP) levels is associated with chronic inflammation and LUTS. In fact, serum CRP levels are significantly higher in patients with OAB and IC than in control individuals. Increased expression of proinflammatory cytokine (IL-1β, IL-6 and TNF-α) and chemokine (IL-8) levels in the serum of IC patients implies not only mast cell activation but also the possible important roles of some other inflammatory mediators in the pathogenesis of BPS/IC [4].

Histological studies showed infiltrates of mast cells, eosinophils, macrophages and T lymphocytes in bladder biopsies of patients with LUTS. BPS/IC bladders have also confirmed the involvement of inflammatory markers in the urothelium and mast cells in the detrusor.

High expression of T and B cell markers, focal lymphoid aggregates in the submucosa, mast cells in the lamina propria and detrusor, as well as high immunoglobulin concentrations in the urine, were found in patients with BPS/IC. When inflammation starts it is a vicious cycle. The chronic exposure to an inflamed organ leads to chronic inflammation and excessive tissue breakdown resulting in tissue degeneration. In fact, chronic suburothelial inflammation has been shown to inhibit normal basal cell proliferation, thus affecting the apical urothelial function. Furthermore, bladder inflammation caused by intravesical irritants leads to acute afferent nerve activity and long-term plasticity, which lowers the threshold for nociceptive and mechanoceptive afferent fibres. Animal models have shown that intravesical mesenchymal stem cell injection into bladders with chemical cystitis ameliorates inflammation and fibrosis, presumably by inhibiting T and B cell proliferation and function [5].

A recent study demonstrated that intravesical GAG replenishment therapy also produces a physiological effect of restoring the permeability barrier of the urothelial cells. Therefore, stopping the exposure of the urine irritants to the suburothelial bladder wall layers decreased the recruitment of inflammatory cells in an acute damaged bladder rat model. However, treatment of urothelial dysfunction cannot be based solely on replacement of defence glycoproteins in the bladder urothelium. Drugs to treat the chronic inflammation and sensory nerve hypersensitivity should also be used.

If the neurogenic inflammation in the dorsal root ganglia can be gradually eliminated through intravesical treatment, the visceral pain and chronic symptomatology can also be relieved.

If the activation and/or irritation of the afferent sensory nerve system in the urinary bladder persists, the bladder inflammation and tissue damage continue and become chronic. This leads to central nervous system sensitisation and chronic symptomatology that is difficult to treat.

## The sensory nerve system

The sensory nerve system in the bladder plays an important role in the pathogenesis of lower urinary tract dysfunction. In fact, there is increasing evidence showing that afferent hyperexcitability is a result of neurogenic bladder inflammation. It has been shown that overexpression of nerve growth factor in mouse urothelium leads to neuronal hyperinnervation, referred somatic pelvic sensitivity, elevated mast cells, and changes in bladder function. Finally, a recent study suggested that BPS/IC might be mediated by factors including changes in the properties of peripheral bladder afferent pathways responding to normally innocuous stimuli.

From the above evidence, it is possible to postulate that the pathophysiology of BPS/IC syndrome as well as LUTS might evolve sequentially by:

1. Urothelial injury (UTI, surgical trauma, chronic overdistention
2. Suburothelial inflammation
3. Chronic inflammatory cell infiltration in the suburothelium
4. Increased inflammatory reaction in the sensory afferents, dorsal horn ganglia, and corresponding spinal cord

## The role of intravesical treatment

Intravesical treatment, also defined as bladder instillation, is a form of therapy and treatment for BPS/IC. Common bladder instillations include intra-vesical lidocaine, hyaluronic acid, dimethyl sulfoxide (DMSO) heparin or chondroitin sulphate as individual or combined therapies.

However, the term “bladder instillation” only defines a way of administering drugs to a patient; the ideal and most effective medications, how long the instilled drugs should be kept in the bladder to ensure the best outcome possible, the correct dose and solution, and the frequency and duration of treatments to be used are still unknown, despite the reduction in urgency noted in various studies.

Various dosing regimens of lidocaine and heparin instillations have been trialled. Parsons et al recently described the use of alkalinized Lidocaine and Heparin for bladder pain [6]. This consists of a single vial of 15-ml solution containing 200 mg of lidocaine and 50,000 units of heparin sodium buffered to pH 7.4 ± 0.2 with sodium bicarbonate (420 mg) in water. . In the published studies it has been proposed that the instillation should be kept in the bladder for a minimum of 30mins and a maximum of 45mins. However, whether or not a longer exposure of the intravesical drugs with the urothelium affects the outcomes still needs to be proven. Finally, various frequency regimes of bladder instillations have been described, ranging from single treatment to 3 times a week for a period of 6 weeks to 12 months.

Lidocaine and heparin seem to be the key drugs to be used intravesically for bladder pain and LUTS by treating the leaking dysfunctional urothelium and a sensory afferent nerve irritability and hypersensitivity [7–13].

Lidocaine is a local anaesthetic and anti-arrhythmic with a short half-life of 1.5 to 3 h. It acts by blocking sensory nerve fibres in the bladder. Alkalinisation of lidocaine encourages stabilisation of a greater percentage of the lidocaine into its non-ionised base form (33% vs 1–2% of unbuffered lidocaine at urine pH 5–6). As the bladder urothelium has greater permeability compared with non-charged ions, alkalinised lidocaine has greater absorption [12].

Other reported benefits of lidocaine include inhibition of histamine release from challenged mast cells, anti-inflammatory effects on eosinophil activity, inhibition of leucocyte adherence, as well as bactericidal effects [12]. These characteristics may explain why some patients have reported prolonged ongoing relief following instillation treatment.

Heparin is a polysaccharide and glycosaminoglycan layer enhancer that is thought to reproduce the activity of native bladder mucosa, reducing the transepithelial migration of solutes such as potassium that could depolarise sensory nerves to stimulate bladder pain, urgency and nocturia [13].

Bladder instillation with heparin alone has been reported to have good clinical improvement from baseline using a six-point scale in 56% of the patients with bladder pain and LUTS [13]. Various dosing levels of heparin have been utilised in trials from 10,000 to 50,000 units; however, optimal dosing has not been determined.

Combined use of intra-vesical alkalinised lidocaine with heparin can provide immediate and prolonged relief from BPS/IC as well as LUTS. Immediate pain relief has been demonstrated in a dose-dependent way with 75% (35 out of 47 patients) and 94% (33 out of 35 patients) reporting significant immediate symptom relief following a 20-min instillation of 40,000 units of heparin with 80 mg or 160 mg lidocaine respectively, the difference in the response rates being statistically significant (*p* < 0.01) [11]. A multi-centre placebo controlled study has shown ongoing efficacy at 12 h following administration, with an average reduction of pain by 21% for placebo control and 42% for active drug (*p* = 0.036) using an 11-point analogue pain score. Global response assessment (GRA) improvement was 13% for control and 50% for the drug (*p* = 0.014). A reduction in urgency symptoms was also noted in 13% following placebo and 35% with the instillation (*p* = 0.0328) [14].

A pilot study of Parson’s solution instillation compared with lidocaine alone showed greater reduction in bladder pain (38% vs 13%, *p* = 0.029) and urgency symptoms (42% vs 8% *p* = 0.003). Greater percentage improvement in GRA at 1 h, which was sustained at 24 h post-treatment, was also noted [6].

Studies using similar preparations of lidocaine – heparin bladder installation also suggested additional benefits with significant improvement to dyspareunia, frequency, voiding volume and nocturia [15].

Reported side effects of a combined instillation affects approximately 30% to 50% of patients and include headache, dizziness, light headedness and bladder or urethral pain. The side effects have been noted at a similar frequency when compared with placebo groups [16]. With doses of 10,000 units of heparin, no increases in activated partial thromboplastin time or prothrombin time were observed [13, 14] when the medication was administered three times a week.

Serum levels of lidocaine at 30 min in a trial administering alkalinised lidocaine at 5 mg/kg were considered safe, with a peak level of 1.3 mcg/ml [17]. A subsequent larger multi-centre, placebo-controlled trial administering 200 mg of alkalinised lidocaine daily for 5 days with a 1-h retention confirmed safe and clinically effective peak serum lidocaine levels of 0.6 mcg/ml at 1.13 h [18] The systemic toxic threshold for lidocaine is considered to be 5 mcg/ml [11] of plasma with a maximum daily dose of 200 mg of lidocaine or 500 mg of lidocaine if combined with adrenaline-containing solutions.

The role of intravesical gentamicin apart from sporadic small studies still needs to be proven. This is also the case for the use of triamcinolone infiltration to Hunner’s ulcers in patients with BPS/IC [19]. Botulinum toxin A intravesical injection has also been shown to improve bladder capacity in BPS/IC and has the highest probability of being the best therapy according to global response and symptom assessment [20, 21].

## Dimethyl sulfoxide

Interest in dimethyl sulfoxide (DMSO) grew after it was found to have local anaesthetic, bacteriostatic and anti-inflammatory properties. Its original urological use involved transdermal application to the supra-pubic region of patients diagnosed with BPS/IC. After showing minimal benefit, DMSO was then used as an intravesical agent with direct application to the bladder wall, with beneficial effects seen in 6 out of 8 patients in initial studies [22]. It is thought to work via reducing interleukin-8-mediated inflammatory responses, decreased NF-κB activation and/or reduced prostaglandin E2 stimulation. Its analgesic effect is thought to be mediated by desensitising nociceptive pathways on the afferent nerves of the lower urinary tract.

Treatment regimens vary, with DMSO being used either in isolation or together with other intravesical medication. The optimal dwell time, length of induction therapy or length of maintenance therapy is unknown. Results from single-arm cohort studies showed efficacy rates of 61–95% in BPS/IC patients who were resistant to medications, hydrodistension and fulguration. The strong garlic odour associated with DMSO makes blinding for a clinical trial challenging. An RCT comparing DMSO with placebo showed a 53% subjective and 93% objective improvement in the DMSO group compared with an 18% subjective and 35% objective improvement in the placebo group [23]. Two randomised controlled trials also showed that DMSO was superior to BCG in the treatment of BPS/IC [24, 25]. Five studies have investigated the use of DMSO as part of a “cocktail” with heparin, hydrocortisone, triamcinolone and/or local anaesthetic. Response rates varied from 61 to 70%; however, overall efficacy rates did not exceed those of DMSO alone. Iyer et al. compared DMSO plus triamcinolone with bupivacaine plus heparin and triamcinolone(B/H/T) [26]. DMSO treatment resulted in a greater percentage of overall improvement and a significant decrease in nocturia episodes compared with B/H/T.

Reported side effects in the literature include increased urgency and dysuria, lethargy, nausea, fever and haematuria, which was thought to be related to transient chemical cystitis; however, these were found to be low in frequency. The use of DMSO in animals has been linked to changes in their eye lenses, but no link has been seen in clinical trials [27]. Long-term follow-up (median 60 months) suggests that DMSO/heparin/hydrocortisone/bupivacaine therapy might appear to be moderately effective, but failure was more frequent in patients with pre-treatment reduced bladder capacity [28, 29].

## Hyaluronic acid and chondroitin sulphate

In chronic inflammatory bladder diseases GAGs such as hyaluronic acid (HA) and chondroitin sulphate (CS) are lost from the bladder lining. Intravesical replacement of these is widely used to treat BPS/IC. Results from 126 patients with BPS/IC treated with weekly HA instillations showed symptom improvement in 85% of patients. 34.5% had symptom recurrence and intravesical treatment was initiated again, whereas the rest stayed symptom free for 5 years. Apart from mild irritative symptoms, no adverse effects were reported [30]. HA used in combination with potassium chloride and sodium chloride showed a 62.5% and 71.48% improvement in pain respectively [31]. When used in combination with lidocaine it may lead to immediate relief of symptoms, with one study showing a reduction in voiding frequency of 67.25% and pain reduction of 70.82% [32].

Another natural proteoglycan present in the GAG layer of bladder epithelium is CS, which has been used as intravesical therapy in BPS/IC. Symptom improvement rates are around 60%, but the concentration of the instillation varied among studies. When combined with hydrodistension, there was a 47% improvement in pain and urinary urgency and a 51.8% reduction in voiding frequency [33].

Studies comparing HA and CS have conflicting results over efficacy; however, combination therapy of HA and CS is available. One regime involves weekly instillations for 8 weeks, then once every 2 weeks for the next 6 months. Results showed a significant reduction in urgency scores and pain scores [34]. Long-term treatment over 3 years showed sustained improvement in bladder function and quality of life; however, this study only assessed 12 patients [35] and further confirmation of long-term outcomes is needed from larger trials. A 53-patient study compared HA alone with HA/CS combined and found no significant difference in symptom improvement between the two groups [36]. Another study showed that when used in combination or CS alone, both regimes had beneficial effects on sexual function [37].

## Other intravesical GAG replenishment drugs

In addition to the above-mentioned drugs, sodium pentosan polysulphate (PPS) has also been used for GAG replenishment in BPS/IC. It is currently the only oral therapy approved for BPS/IC in the USA, but in Europe its use is off label. Studies have suggested that it might result in significant improvements in pain and urgency. There is also evidence that oral PPS combined with an intravesical cocktail of heparin and hydrocortisone leads to a good to excellent response at 1, 3 and 6 months in 91.3% of patients (Taneja, personal communication).

Two studies have investigated intravesical PPS use for 3 months. Compared with placebo, one study showed significant improvement in bladder capacity and night-time frequency, but no change in daytime frequency or volume of first desire to void [38]. The other open label study showed improvement in the VAS for quality of life after 20 instillations during a 10-week period [39] However, side effects, such as diarrhoea, abdominal pain and rectal bleeding have been reported and one study noted alopecia in 5% of patients. In view of these side effects PPS is recommended as second-line therapy for BPS/IC [33]. Most recently, a possible relationship between long-term PPS exposure and development of pigmentary maculopathy has been suggested. More research is necessary to further elucidate a causal relationship, but caution is suggested when prescribing PPS especially in patients with pre-existing retinal conditions [40].

A double-blind placebo-controlled trial demonstrated the safety and efficacy of the use of a combined therapy of intravesical and oral PPS for the treatment of moderate and severe IC patients. The combined use of intravesical PPS and oral PPS has been shown to enhance the proliferation of the GAG layer of the bladder, to produce greater relief and return to normal protective coating when maintained with oral PPS [41].

## Conclusions

Chronic LUTS can be considered the result of a progressive disease that evolves from early-stage to late-stage bladder conditions. Insult to the visceral organ initiates an inflammatory process causing urothelial dysfunction in the bladder. The inflammatory reaction proceeds along the sensory nerves in the dorsal root ganglion, as well as the sacral cord. The sensory impulse also ascends to the corresponding cortical gyrus. Patients might have an early inflammatory reaction and produce LUTS, including bladder pain, urgency, frequency, etc. If the insult does not continue, the inflammation resolves and patients may have relief after treatment of symptoms. However, if the bladder insult continues, the inflammatory reaction increases to a higher level, causing permanent inflammation and chronic refractory symptoms. This highlights the important role of intravesical GAG replenishment therapy in the management of patients with BPS/IC or bladder inflammation.

Bladder instillations also have the advantage of targeting the bladder directly, thus optimising the efficacy of the drugs used and reducing their side effects profile. Despite the data reported in the literature still being scarce, the use of drugs with anti-inflammatory properties, able to desensitise hypersensitive nerves, as well as restoring the permeability of damaged urothelial cells, may be a sensible approach to all kinds of LUTS secondary to bladder inflammation, and not just bladder pain. An adequately buffered solution should also be considered to reduce the event of drug precipitation, which can occur when heparin and lidocaine are mixed separately without proper alkalinisation [9]. Benefits of a combined heparin and alkalinised bladder instillation has been demonstrated, with prolonged relief beyond the half-life of lidocaine described. Combination therapy may be acting to treat the inflammation of BPS/IC through the anti-inflammatory and bladder mucosa-stabilising properties of lidocaine and heparin, as well as providing immediate pain relief through the anaesthetic properties of lidocaine. However, this assumption needs to be scientifically proven by large randomised control trials. These studies are also required to define the ideal and most effective medications, how long the instilled drugs should be kept in the bladder to ensure the best outcome possible, the correct dose and solution to avoid the precipitation of the drugs used, as well as the frequency and duration of treatments. There are a wide variety of both single-agent and combination-agent intravesical treatments in use and there needs to be adequate assessment through large clinical trials with high methodological quality addressing appropriate patient-reported outcomes [42]. Despite the attempts of various investigators that have already been proposed to make the best bladder instillation solution by adding different drugs together, there is still a lack of scientific evidence supporting what drug or drugs should be used to treat bladder pain and why not LUTS in general.
